# Complex floral traits shape pollinator attraction to ornamental plants

**DOI:** 10.1093/aob/mcac082

**Published:** 2022-06-22

**Authors:** E Erickson, R R Junker, J G Ali, N McCartney, H M Patch, C M Grozinger

**Affiliations:** Department of Entomology, Center for Pollinator Research, Huck Institutes of the Life Sciences, Pennsylvania State University, ASI Building University Park, PA, USA; Evolutionary Ecology of Plants, Department of Biology, University of Marburg, 35043 Marburg, Germany; Department of Environment and Biodiversity, University of Salzburg, Salzburg, Austria; Department of Entomology, Center for Pollinator Research, Huck Institutes of the Life Sciences, Pennsylvania State University, ASI Building University Park, PA, USA; Department of Entomology, Center for Pollinator Research, Huck Institutes of the Life Sciences, Pennsylvania State University, ASI Building University Park, PA, USA; Department of Entomology, Center for Pollinator Research, Huck Institutes of the Life Sciences, Pennsylvania State University, ASI Building University Park, PA, USA; Department of Entomology, Center for Pollinator Research, Huck Institutes of the Life Sciences, Pennsylvania State University, ASI Building University Park, PA, USA

**Keywords:** *Salvia nemorosa*, *Rudbeckia* spp, *Echinacea* spp, *Nepeta* spp, *Agastache* spp, pollinators, plant–pollinator interactions, pollination syndromes, ornamentals, bees, floral traits

## Abstract

**Background and Aims:**

Ornamental flowering plant species are often used in managed greenspaces to attract and support pollinator populations. In natural systems, selection by pollinators is hypothesized to result in convergent multimodal floral phenotypes that are more attractive to specific pollinator taxa. In contrast, ornamental cultivars are bred via artificial selection by humans, and exhibit diverse and distinct phenotypes. Despite their prevalence in managed habitats, the influence of cultivar phenotypic variation on plant attractiveness to pollinator taxa is not well resolved.

**Methods:**

We used a combination of field and behavioural assays to evaluate how variation in floral visual, chemical and nutritional traits impacted overall attractiveness and visitation by pollinator taxonomic groups and bee species to 25 cultivars of five herbaceous perennial ornamental plant genera.

**Key results:**

Despite significant phenotypic variation, cultivars tended to attract a broad range of pollinator species. Nonetheless, at the level of insect order (bee, fly, butterfly, beetle), attraction was generally modulated by traits consistent with the pollination syndrome hypothesis. At the level of bee species, the relative influence of traits on visitation varied across plant genera, with some floral phenotypes leading to a broadening of the visitor community, and others leading to exclusion of visitation by certain bee species.

**Conclusions:**

Our results demonstrate how pollinator choice is mediated by complex multimodal floral signals. Importantly, the traits that had the greatest and most consistent effect on regulating pollinator attraction were those that are commonly selected for in cultivar development. Though variation among cultivars in floral traits may limit the pollinator community by excluding certain species, it may also encourage interactions with generalist taxa to support pollinator diversity in managed landscapes.

## INTRODUCTION

Human land-use has led to a global reduction in pollinator habitat and decline of high-quality foraging resources (Potts *et al.*, 2010; Wagner *et al.*, 2021). To mitigate these losses and support pollinator populations, there has been increased public interest in creating pollinator habitat in managed landscapes such as gardens, municipal parks and seminatural habitats (Campbell *et al.*, 2017; Majewska and Altizer, 2020). Due to commercial availability and market preferences (Campbell *et al.*, 2017), many of the flowering plant species used in these domestic environments are ornamental cultivars (Frankie *et al.*, 2005; Burghardt *et al.*, 2009). Certain cultivars have the potential to support pollinator communities, particularly in the context of highly modified urban and suburban landscapes (Garbuzov and Ratnieks, 2014; Rollings and Goulson, 2019; Erickson *et al.*, 2020; Sponsler *et al.*, 2020). However, ornamental cultivars result from a long history of breeding through hybridization and artificial selection (Horn, 2002; Wilde *et al.*, 2015) with human preference as the primary selection pressure. Thus, these cultivars represent a range of floral phenotypes that are often distinct from parent wild types (Garbuzov and Ratnieks, 2015; Erickson *et al.*, 2020). Despite their prevalence in anthropogenic landscapes and managed pollinator habitat, the mechanisms of pollinator community attraction to these varieties are not well understood.

Angiosperms are phenotypically diverse (Armbruster, 2014), and the complex structural, visual, chemical and nutritional traits of flowers are important determinants of pollinator foraging behaviour (Leonard *et al.*, 2011; Willmer, 2011; Junker and Parachnowitsch, 2015). Pollinator taxa (e.g. bees, flies, butterflies and beetles) vary in their perception of and preference for suites of floral traits based on natural history, sensory abilities, learned associations and cognition (Chittka and Raine, 2006; Raine and Chittka, 2007; Willmer, 2011). Pollinator species’ behaviour and morphology influence their ability to access floral resources and serve as effective pollen vectors (Wang *et al.*, 2017). Bee taxa, in particular, exhibit an impressive diversity of foraging behaviours and are especially variable in their preference for floral resources (Danforth *et al.*, 2019). In co-evolved plant–pollinator communities, reciprocal adaptation of plants and pollinators has led to evolution of distinct floral phenotypes which attract the dominant or most effective pollinator taxonomic group (Willmer, 2011). In mutualistic plant–pollinator relationships, floral visual and olfactory cues – or ‘advertisements’ – are hypothesized to be generally honest signals of nutritional rewards (primarily pollen and nectar) to visiting pollinators (Wright and Schiestl, 2009; Schiestl and Johnson, 2013; Knauer and Schiestl, 2015). Honest floral signals facilitate pollinator learning (Knauer and Schiestl, 2015), which increases forager constancy and efficiency (Ortiz *et al.*, 2021), even in highly opportunistic foragers.

Horticultural plant breeding results in significant variation in traits such as colour, morphology and nutritional reward among cultivars of the same genus or even species (Comba *et al.*, 1999; Corbet *et al.*, 2001; Garbuzov and Ratnieks, 2014, 2015; Erickson *et al.*, 2020, 2021). Notably, some floral traits that are relevant to pollinator attraction, such as floral display size (Thompson, 2001), may also be influenced by cultural practices (e.g. pruning, planting design) as well as breeding. Since floral phenotypes in these cultivars have been selected by humans and not pollinators, floral advertisements and rewards may be uncoupled from pollinator preference. In extreme cases, ornamental plant breeding produces cultivars that are valueless or entirely inaccessible to pollinators, such as doubled varieties, or those that no longer offer nutritional rewards (Comba *et al.*, 1999; Erickson *et al.*, 2020). Nonetheless, many ornamental cultivars do have the capacity to attract and support pollinators (Garbuzov and Ratnieks, 2014, 2015; Erickson *et al.*, 2020, 2021). However, several field studies have demonstrated significant variation in the overall and relative attractiveness to pollinator taxa among cultivars of single species (Garbuzov and Ratnieks, 2014; Erickson *et al.*, 2020, 2021), indicating a potential interaction between floral phenotype and overall plant attractiveness.

To date, many studies on pollinator mediated selection on floral traits have focused on visitation by single species and primarily in specialized systems (Burger *et al.*, 2010; Byers *et al.*, 2014). However, generalization is ubiquitous in ecological communities (Danforth *et al.*, 2019) and interactions between plants and pollinators exhibit a high degree of stochasticity and are rarely obligate (Willmer, 2011). Ornamental plants often attract generalized pollinators (Erickson *et al.*, 2020, 2021). As they exhibit a high degree of variation in floral phenotype both within and across genera, these cultivars offer a powerful system to test the attractiveness of diverse floral traits to a generalized pollinator community. Understanding how pollinator taxa and communities respond to variation in floral traits is critical for untangling the role of generalist species or taxonomic groups as agents of selection (Waser *et al.*, 2009; Gervasi and Schiestl, 2017).

Ornamental cultivars within genera often vary in a few measurable traits and probably have limited intracultivar genetic variability due to propagation methods. Thus, they present an opportunity to test the relative influence of single and multimodal floral traits on mediating pollinator species and community foraging behaviour in the context of a whole floral display. Floral advertisements are complex signals, and multiple traits often function synergistically or antagonistically upon multiple pollinator sensory modalities (hereafter referred to as ‘multimodal traits’) (Williams *et al.*, 2010; Junker and Parachnowitsch, 2015; Russell *et al.*, 2018). Furthermore, floral visual, chemical and nutritional traits can be pleiotropic at a genetic level (Smith, 2016), meaning that selection on one trait may influence the expression of another trait, which in turn influences pollinator attraction. Thus, there is a clear need for additional studies that can further elucidate the relationship between pollinator choice and floral traits against the backdrop of a multimodal phenotype (Leonard *et al.*, 2011).

In this field and lab-based study, we use multivariate analyses to test how key floral traits (floral morphology, colour, scent, pollen and nectar rewards) of 25 ornamental plant cultivars across five herbaceous perennial plant genera influence pollinator visitation. For the first level of analysis, we examined how floral phenotypic traits across plant genera explained broad patterns of bee, fly, beetle and butterfly visitation. Because bees were found to visit all 25 cultivars and have sophisticated sensory systems that vary across species, we next investigated how trait variation among cultivars within plant genera structured floral preferences of bee species.

## METHODS

### Plant selection

Plants were selected to reflect variation in cultivar phenotype both among and within genera of commercially popular taxa. The five plant taxa used in this study were: *Salvia nemorosa*, *Nepeta* spp., *Echinacea* spp., *Rudbeckia* spp. and *Agastache* spp. The former two taxa are non-native to the Nearctic region while the latter three are native to this region. All taxa had significant wholesale value in the North American market and thus can be assumed to be widely used in home garden and landscaping applications (USDA—NASS, 2014). Plants were purchased from North Creek Nursery (Oxford, PA), Creek Hill Nursery (Leola, PA), Russell Gardens (Southampton, PA), Morningsun Farm (Vacaville, CA) and Bluestone Perennials (Madison, OH). Original plants were purchased in 2017 and were replaced annually as needed after assessing overwinter survival. Plants were never treated with neonicotinoid insecticides throughout the study, and glasshouse pest outbreaks were managed with insecticidal soap. Care was taken to ensure that no collections were done on plants immediately following treatment. We cannot account for how the plants were managed at all nurseries prior to our acquiring them (see Supporting Data for details on plug treatment at the nursery where most plant material was sourced). However, based on the time span between any potential application of any pesticides at the source nursery and when our data were collected, we expect that pollinator exposure in the field to any chemicals that would confound our results would be negligible (Cowles and Eitzer, 2017; Mach *et al.*, 2018).

### Pollinator preference assessments

As environmental context and ecological community dynamics can shape plant attractiveness to pollinators (Geslin *et al.*, 2013; Kantsa *et al.*, 2018), pollinator foraging preferences were assessed using both field collections and lab-based choice assays.

### Field collections

The data used in these analyses were a subset of a data set used in a previous study (see Erickson *et al.*, 2021 for details). Briefly, field data were collected across two independent sites in 2019 at the Penn State Russell E. Larson Agricultural Research Center in Pine Grove Furnace, PA (Site 1 = 40.704634, −77.973045, Site 2 = 40.712329, −77.933609). Due to poor soil conditions, plants at Site 1 were in pots whereas plants at Site 2 were planted directly in the ground. All potted plants were grown using Sungro MetroMix 830 (Agawam, MA, USA) media. Plants were fertilized at both sites in May 2019 to genus-specific levels with Osmocote Plus 7.5-g Tablets 15-8-11 (Scott’s Miracle-Gro, Marysville, OH, USA). Plants were arranged in a randomized complete block design with four blocks per plot and one replicate of each cultivar per block, for a total of 100 plants at each site. Within each block, plants were spaced 1 m apart and blocks 1.5 m apart.

All insect flower visitors to each plant replicate were collected for 5 min every other week from May 23 to September 23, 2019. For each sampling event, specimens were collected from each replicate once in the morning (09:00–13:00 h) and once in the afternoon (13:01–17:00 h) using an insect vacuum (Bioquip, Rancho Dominguez, CA, USA). Collections were performed in sets of two down the rows of the plot and the starting point alternated for each collection event. Specimens were euthanized in the field using dry ice then transferred to individual vials and stored in the laboratory at −20 °C until they could be processed and identified. Specimens were pinned and bees identified to species with assistance from S. Droege (USGS Patuxent Wildlife Research Center).

### Lab-based choice assays

Cultivar choice and foraging behaviour of *Bombus impatiens* foragers on cultivars were recorded in the laboratory. Experiments were conducted using two custom built foraging arenas as in Russell *et al.* (2018) (see Supplementary Information Figure S1 for specifications). *Bombus impatiens* colonies were purchased from Koppert Biological Systems (Howell, MI, USA). Colonies were only included in choice assays if they were in the social phase of their life cycle – that is, they had not initiated gyne production and had few males (Amsalem *et al.*, 2015). When not in use, colonies were stored in an incubator at 23 °C and maintained on 50 % (w/w) sugar syrup and honey bee-collected pollen according to standard practices (Velthuis and van Doorn, 2006). All colonies used in this study were flower naive – meaning they had never been previously exposed to real flowers or floral advertisements (ex. floral scent, colour). See Supplementary Information Table S1 for details on colony-level replication.

Before testing, colonies were attached to the training arena via a 12 × 5-cm plastic tube to allow foragers to acclimate. Each training arena included two feeders with 20 % (w/w) sucrose solution and a tray of honey bee-collected pollen. Feeders were painted black to avoid foragers associating colours found in test flowers with reward (Muth *et al.*, 2015). Foragers were allowed to pass freely between the colony and the training arena for a minimum of 24 h and a maximum of 7 d before testing. Feeders were replenished daily to ensure consistent availability of food.

A separate arena was used to test forager preference for cultivars. Experimental trials were performed for within-individual plant genera, and individual foragers were allowed to choose between the five cultivars of either *Agastache* spp., *Echinacea* spp. and *Rudbeckia* spp. In each trial, three replicate inflorescences for each cultivar (grown in the glasshouse) were placed in water picks and mounted vertically on the long side of the arena. The source plant for these inflorescences was randomized across trials. Care was taken to select only inflorescences that were still producing nectar and/or pollen rewards and appeared healthy. Individual inflorescences were replaced after a successful foraging event or every 2 h if not visited to ensure freshness and presence of consistent floral scent and reward (Morrant *et al.*, 2009; Ao *et al.*, 2013). At the start of each trial, inflorescences were rearranged to prevent foragers from associating particular areas of the arena with rewards, and the locations of each flower were recorded (Raine and Chittka, 2007).

To test how cultivar phenotype influenced initial preferences, individual foragers that had not been previously tested, hereafter referred to as ‘naive foragers’, were collected from the training arena using an insect vacuum (Bioquip). Naive foragers were introduced to the training arena from the vacuum’s clear plastic collection canister. The testing trial started once the single forager entered the arena. Foragers were allowed to visit three inflorescences before being collected and removed from the arena. For each trial, time to initiate foraging (the time between entering the arena and initiating foraging on the first cultivar), cultivar choice (which cultivars the bee successfully foraged on), duration of foraging (length of time spent foraging on each of the three inflorescences), time between foraging events (interval between foraging events), and pollen and nectar collecting behaviour were recorded. Pollen foraging behaviour was characterized by the bee moving erratically over the anthers and brushing pollen onto the corbicula, while nectar foraging behaviour was characterized by the bee probing the corolla with its proboscis extended. Foragers that did not fly within 5 min or that flew but did not explore flowers (exploration being classified as a bee flying within ~5 cm of the flower and exhibiting signs of recognizing the flower such as circling or directional flight but ultimately not foraging) within 10 min were removed from trials.

Next, we used the successful foragers from the initial trial – hereafter referred to as ‘experienced foragers’ – to test whether floral visual and olfactory advertisements were a sufficiently honest signal of nutritional rewards (Wright and Schiestl, 2009; Knauer and Schiestl, 2015). Experienced foragers were held in isolation for 1 h in a plastic collection canister before reintroducing them for an ‘experienced’ trial and recording the same metrics. For each plant genus, there were between three and seven replicate trials per colony (most had six replicates) and either four or five colonies (see Supplementary Information for details of the data sets generated). All foragers that were introduced to the test arena, regardless of whether they had a successful foraging bout, were killed promptly to avoid their nestmates from gaining information on resource quality or floral cues (Molet *et al.*, 2009).

### Assessment of plant traits

Two additional replicates of each cultivar were grown in the glasshouse in 2018, 2019 and 2020 for collection of data on floral traits. Additionally, plants that had been housed outdoors in 2018 and 2019 and used for field data collections were brought into the glasshouse for use in choice assays and traits collections. All glasshouse plants were replaced in 2018. In 2019, all glasshouse plants were vernalized outdoors for reuse the following season. See Supplementary Information Table S2 for details on which plant sets were used for collection of different trait data, and associated number of replicates.

### Floral area and plant height

**Fig. 1.**
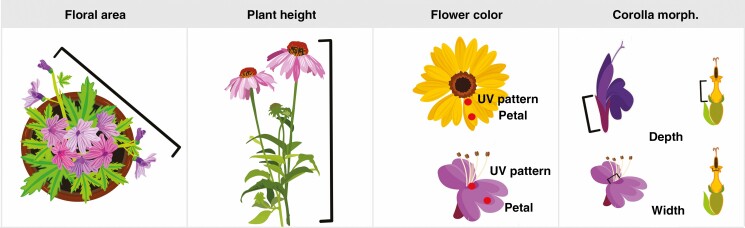
Diagram of trait collections for floral area, plant height, flower colour and corolla morphology.

Total floral display area was measured every other week on each plant replicate in the field throughout the season in 2019 by photographing a single plant from above with a metre stick held at the level of the crown of the floral display. These photos were processed in Adobe Photoshop (22.3.0) by setting a unique measurement scale in pixels based on the metre stick and then selecting all flowers, based on colour, to calculate the total area in in bloom (Fig. 1). Additionally, the number of inflorescences per plant with at least 10 % of flowers or florets in anthesis was measured for each photo and was used to standardize visitor abundance measures.

Plant height was measured from the ground to the crown of the plant for each replicate in the field once at peak maturity. For all plant taxa in this study, vertical growth was minimal after the initiation of flowering (Fig. 1).

### Floral colour

Flower colour reflectance was measured for five technical replicate inflorescences from each of the two biological replicates (different plants) collected from the glasshouse using an Ocean Optics USB2000 + UV-VIS spectrometer and DH-2000BAL light source with a 200-μm reflectance probe (Ocean Optics, Winter Park, FL, USA). Before beginning collections, the spectrometer was first standardized using a white diffuse reflectance standard (Ocean Optics). Collections were all done indoors with an overhead LED as the sole ambient light source. For each flower, measurements were performed in triplicate at two points (petals and the mouth of the corolla) by holding the probe at a 90° angle 1 cm from the petal surface and then averaging measurements from for each point for analysis. For all measurements, the spectrometer was set to a 0.1-s integration time with 10 scans to average, and a boxcar width of 0. Petal measurements were used to represent flower colour for analyses, and reflectance measures at both points were used to detect the presence of ultraviolet patterns (Chittka *et al.*, 1994; Horth *et al.*, 2014).

Reflectance data were processed using Ocean View software (Ocean Optics, Version 4.1). Reflectance measures gathered between wavelengths of 300 and 700 nm were averaged across consecutive 5-nm segments for each replicate. Pollinators vary widely in their colour perception, so these raw spectral data were used for ordination analyses to prevent biasing results towards any particular taxa (Shrestha *et al.*, 2013). For linear models, where a single value is needed for analysis, hue angle was calculated for each replicate based on trichromatic human colour space using the ‘pavo2’ (Maia *et al.*, 2019) package in R. The presence of ultraviolet patterns, such as nectar guides (Leonard and Papaj 2011), was checked for each cultivar by identifying whether any measured wavelengths within 300–400 nm had reflectance values of at least 10 % intensity of the peak value (Chittka *et al.*, 1994). A cultivar was considered to have ultraviolet pattern present if wavelengths in the UV spectrum were recorded in at least two replicate flowers (Fig. 1).

### Floral scent

Headspace volatile organic compound (VOC) emissions were measured on glasshouse plants using dynamic headspace sampling. Individual intact inflorescences were enclosed with a nylon oven bag (Reynolds Consumer Products, Lake Forest, IL, USA) that had been baked for 24 h at 80 °C to remove any polymer VOCs (Stewart-Jones and Poppy, 2006) and secured at the stem with twist ties. VOCs were collected over 8 h from around 09:00 to 17:00 h at a flow rate of 250 mL min^–1^ over HayeSepQ filter traps (Sigma Aldrich, USA). Passive incoming air was purified over activated charcoal. VOCs were collected on single inflorescences from three biological replicates (individual plants) per cultivar.

Additional collections were performed to compare headspace VOCs on cut vs. intact flowers to determine whether cut flowers (used in behavioural assays) had altered profiles. The rate of VOC emissions and VOC diversity between glasshouse and cut flower collections were compared using ANOVA and the number and identity of unique compounds in cut vs. intact flowers for each cultivar was assessed (presence/absence). See SUpplementary Information for further details on these analyses.

Following collections, measured inflorescences were cut and dried at 37 °C for 48 h to then be weighed. Dry tissue weight was used to standardize emission rates. VOCs were eluted from filter traps using 150 µL dichloromethane and 5 µL of an internal nonyl acetate standard and stored at −80 °C until analysis. Samples were analysed using an Agilent 7890B gas chromatograph and 5977B mass spectrometer held at 250 °C. For each sample, 1 µL was injected and run through a column (HP-5MSUI, 30 m, 0.25 mm id, 0.25 μm, Agilent, USA) held at 40 °C. Compounds were classified based on Knudsen *et al.* 2006. See Supplementary Information for information on compound identification. The log sum emission rate (ng g^–1^ h^–1^), Simpson’s diversity (1/*D*) of all emitted compounds and the proportional emissions rate of each compound (the latter two measures based on non-log-transformed raw values) were calculated for each cultivar for statistical analyses.

### Nectar

Nectar was collected from five florets per inflorescence at five replicate inflorescences per plant and on two replicates (individual plants) for each cultivar at both Site 1 in 2019 and Site 2 in 2018 and 2019, for a total of 10 total replicates per cultivar per site, using 1- to 10-µL glass microcapillary tubes. Plants were covered with fine mesh exclusion bags in the field for 24 h prior to collections to ensure adequate availability of nectar reward. Collections were only performed on healthy plants between 09:00 and 17:00 h. Nectar volume was estimated for each flower based on the height of the liquid in each capillary tube, measured using digital calipers (Mitutoyo 500-196-30).

Variation in nectar quantity and quality between sampling locations and years (for Site 2) was assessed using a linear mixed effects model with site or year and cultivar as fixed effects and the biological replicate identifier as the random effect (see Supplementary Information for results).

To assess nectar sugar composition, the cumulative nectar from a single inflorescence (five florets) was then expelled onto filter paper using a rubber bulb and samples were stored at −20 °C until analysis. Nectar volume per floret was estimated by averaging the cumulative nectar volume per inflorescence by the number of florets collected from. Nectar samples were washed from the filter paper using 20 µL to 1 mL of water (Morrant *et al.*, 2009) (approximately a 1:100 volume dilution, which was accounted for in the final analysis) and then the eluted samples were cleaned for analysis using 0.45-μm spin filters in 1.5-mL microcentrifuge tubes, with centrifugation at 13000 r.p.m. for 10 min (Power *et al.*, 2018). The fructose and glucose (monosaccharide) and sucrose (disaccharide) concentrations and the total concentration of the diluted nectar samples were analysed at the CSL Behring Fermentation Facility using a Thermo Scientific Vanquish UHPLC system (Waltham, MA, USA) with a 50 × 2.1-mm Thermo Scientific Hypersil Gold amino HPLC column for 1.9-μm particle size and a Refractomax 521 Refractive Index (RI) Detector. Due to column ageing, methods had to be adjusted between runs; however, a two-way ANOVA and Tukey post-hoc test confirmed no significant differences between methods in the final results (data not shown). In total, 2.5–3 µL of sample was run through the UHPLC in a mobile phase of either 80 % acetonitrile (ACN)/20 % water or 85% ACN/15% water at a flow rate of either 0.5 or 1 mL min^–1^. The RI Detectorwas held at 35 °C for all runs and the column compartment was held at either 40 or 35 °C (see Supplementary Information for specific methods). In cases where a single sample was analysed multiple times using different methods, values were averaged.

### Pollen

Pollen samples were collected by clipping mature anthers from five inflorescences from two replicates of each cultivar at each field site and two replicates of each cultivar in the glasshouse. Plants in the field were covered for 24 h with a fine mesh exclusion bag before collections. Samples were stored at −20 °C until analysis. See Supplementary Material for details on pollen extraction from anthers.

Pollen protein content was measured using a Bradford assay following protocols from Vaudo *et al.* (2016) and the amount of reagent used was adjusted depending on sample weight (see Supporting Data). Total protein concentration in the sample was measured using a SpectraMax® 190 Microplate Reader (Molecular Devices, San Jose, CA, USA) and standardized using total sample mass. Due to variation in results and abiotic factors, only samples collected in the field and with at least 0.007 mg of tissue were ultimately included in statistical analyses on pollen protein.

Total pollen amount for each replicate was estimated by calculating the mass of the pollen sample for each cultivar replicate.

### Corolla morphology

Corolla depth (from base to mouth) and width (diameter at mouth) was measured on glasshouse plants using adjustable digital calipers (Mitutoyo 500-196-30), as in Courcelles *et al.* (2013). Twenty-five replicates of each measurement were taken on two biological replicates per cultivar (Fig. 1).

### Statistical analysis

All quantitative analyses were performed in the R statistical software version 4.0.3.

### Phenotypic variation among cultivars and genera

The difference among cultivars (predictor variable) in individual floral trait states (response variables) was estimated using separate linear mixed effects models (LMMs) or linear models (LMs) (see Supplementary Information for model information) and comparisons among cultivars were calculated using Tukey post-hoc tests. Model fits were assessed using analysis of residuals.

### Floral traits that predict overall abundance of pollinators in field assays

A generalized linear model (GLM) fit to a Gamma distribution was used to estimate which traits explained the overall attractiveness of a cultivar to pollinators in the field. The response variable for this analysis was ‘mean abundance of all visitors/5 min’ to each plant replicate in the field. The predictor variables were the mean values for individual floral traits measurements for each cultivar, except for floral display area and plant height, which were averaged for each cultivar at each site, to account for variation in growth patterns. Based on analysis of residuals, the predictor variables plant height, floral area and mean VOC emissions were log-transformed. Model fit was assessed using analysis of residuals. In addition, residuals were checked using the DHARMa package to check quantiles and outliers (Hartig 2021) (see Supplementary Information Fig. S2 for analysis of residuals).

### Determining how floral traits across plant genera influence relative attraction of pollinator taxonomic groups in field assays

Mean abundance of visitors per inflorescence in 5 min from each pollinator taxonomic group (Anthophila, Coleoptera, Diptera, Lepidoptera) was calculated for each plant replicate in the field. To avoid ‘rare’ groups that may confound analyses, visitors from a taxonomic group that were collected fewer than five times at a cultivar across the whole season were removed.

Non-metric multi-dimensional scaling (NMDS) was used for the following analyses as this method allows the user to specify the similarity function. Separate ordinations were used to determine assembly of plant taxa in traits space for each set of traits. The separation of plant taxa in traits space was analysed using a PERMANOVA. For all PERMANOVAs, 999 permutations were used. Plant display size, based on plant height and floral display area, colour (binned reflectance values), corolla morphology, headspace VOCs emission rate and inverse Simpson’s diversity (1/*D* – used in place of raw VOC compound number to account for convergence errors due to variable scaling), and nectar and pollen qualities were analysed separately using a Euclidean dissimilarity matrix. As outlier observations receive disproportionate weight in a Euclidean dissimilarity matrix, all of the aforementioned trait measurements were log-transformed prior to ordination, with the exception of nectar monosaccharide percentage and colour as these values are on a discrete scale. A Bray–Curtis dissimilarity matrix, which can handle zero values, was used to analyse proportional emission rates of compounds. For traits collected in the field (plant height, floral display area), site was included as a fixed effect for PERMANOVAs (data not shown). For traits that were collected with repeated measures on biological replicates (colour, morphology, nectar, pollen), additional PERMANOVAs were conducted to account for variation within replicates (or, random effects) with the fixed effect of replicate and plant identifier set as the strata argument (Theodorou *et al.*, 2020) (see Supplementary Information for results on these analyses). Distance-based analyses may be biased by data with high variance and heterogeneity (Warton *et al.*, 2012), and so ultimately only ordinations with a PERMANOVA statistic (*R*^2^) of at least 0.15 were included in analyses. To visualize interactions between floral traits and pollinator preference, environmental vectors indicating the direction and strength of pollinator taxonomic group abundance in phenotypic space were fit to each floral trait ordination using the ‘vegan’ package (Oksanen *et al.*, 2020).

### Inter-cultivar variation in floral phenotypic traits and bee species’ visitation in field assays

How variation among cultivars within genera in floral phenotype impacts relative attractiveness and accessibility of a cultivar to pollinators was assessed at the pollinator species level. As Anthophila visitors were ubiquitous across cultivars and their natural history and functional traits are well resolved, these analyses were performed only for bee species.

The mean abundance of visitors/inflorescence/5 min to cultivars within bee species was calculated for each plant replicate across the growing season. Bee species with fewer than four specimens collected per cultivar were excluded from the analysis. The remaining species were then categorized based on functional traits that may be relevant to foraging behaviour in this system. The bee functional traits used for this study were intertegular distance (ITD), which is a proxy for body size (Normandin *et al.*, 2017; Laha *et al.*, 2020), tongue length, diet breadth, phenology and pollen transport system. The latter four traits were assessed for each species using previously published literature (Bartomeus *et al.*, 2013; Normandin *et al.*, 2017). ITD was measured on five randomly selected pinned specimens per bee species from this field study and then these measurements were averaged for each species. ITD measures were then converted to categorical data for analyses using 2 mm as the range of sizes included within each category that was relative to our data (ex. ‘small’) (see Supplementary Information Table S3 for classifications).

The mean abundance of bee visitors/inflorescence/5 min based on functional traits was calculated for each plant replicate in the field.

NMDS ordinations of bee species preference, based on abundance of individuals within each species with shared functional traits collected on each plant replicate, were performed for the five cultivars in each plant genus using a Bray–Curtis dissimilarity matrix. Dissimilarity of cultivars in bee preference space was assessed using PERMANOVA with site set for the strata argument.

Additional NMDS ordinations of cultivar floral functional traits (ex. colour, morphology) were performed within plant genera and the dissimilarity of cultivars in traits space was assessed using PERMANOVA. Mantel tests (with a Spearman correlation coefficient) were performed to assess whether dissimilarity of cultivars based on floral functional traits were correlated with dissimilarity of cultivars based on bee visitation, and when relevant to the trait, abundance of visitors by bee functional trait. Mantel tests with a significant correlation coefficient (*R*) below 0.15 were considered to have weak associations between preference and trait matrices (Johnson, 2013).

### Validation of bee species preference to cultivars in laboratory choice assays

Community and environmental context can shape pollinator visitation to flowers (Cusser *et al.*, 2019). To ensure that the patterns of bee species preference for cultivars used in our analyses were not simply an artifact of these variables, preferences of *B. impatiens* foragers to cultivars in the field and in laboratory choice assays were compared.

The probability of a cultivar being visited by a *B. impatiens* forager was modelled separately for lab and field assays and within plant genera. Generalized linear mixed models (GLMMs) were used to model field data, with individual plant replicate included as a random effect and number of inflorescences with flowers in anthesis and cultivar as predictor variables. Due to model singularity when choice assay data were run as GLMMs with colony as a mixed effect, GLMs were used. However, additional analyses (not shown) were run to confirm that colony did not have a significant effect on choice assay model results. Both field and choice assay models were fit to a binomial distribution. The estimated marginal means (EMMs) of the probability that a forager visited a cultivar were extracted from each model (Lenth 2020).

Within choice assays, the probability of a cultivar being the first or second choice and the probability of a cultivar being the first choice in a naive vs. experienced trial was compared using EMMs extracted from a binomial GLM and a Tukey post-hoc test (data not shown).

## RESULTS

### Phenotypic variation of cultivars

**Fig. 2.**
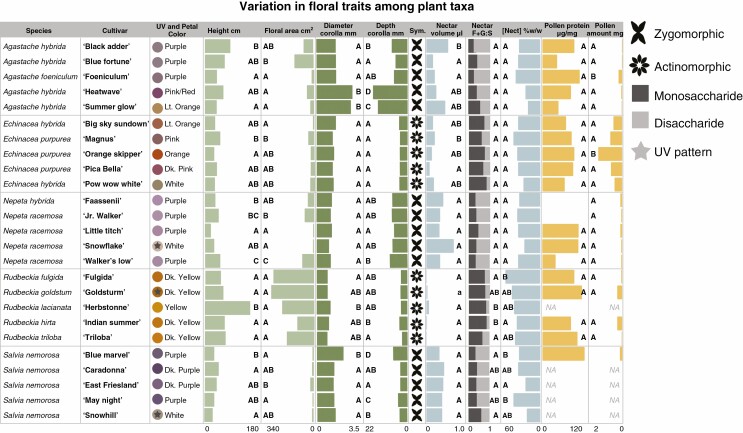
Means of trait measurements for each cultivar based on emmeans from LM and LMM regression analyses. Letters denote significant differences among cultivars within plant genera based on Tukey post-hoc test results at *P* < 0.05. Cultivars within all plant genera vary in some traits, with the greatest variation in flower colour, plant size and corolla morphology. Cultivars with a star over the colour point are those where a UV patten was detected in reflectance measures. ‘Sym’ refers to flower symmetry (either zygomorphic or actinomorphic). Nectar F + G:S refers to the nectar monosaccharide to disaccharide ratios. Pollen amount is measured by weighing dry tissue mass of collected samples before analysis.

Within genera, cultivars exhibited significant variation in some floral phenotypic traits but not others (results are summarized in Fig. 2). All cultivars varied in petal colour (visualized by hue angle in human colour space), and ultraviolet pattern was detected in three cultivars in three separate genera. Cultivars varied significantly in at least one trait pertaining to plant size (area and height). Cultivars of all genera except *Echinacea* spp. differed from one another in at least one trait related to corolla morphology, with corolla depth being the most variable. All cultivars of *Agastache* spp., *Nepeta* spp. and *S. nemorosa* were zygomorphic while those of *Echinacea* spp. and *Rudbeckia* spp. were actinomorphic.

There was significant variability among cultivars of *Agastache* spp. and *Echinacea* spp. in nectar volume and among cultivars of *Rudbeckia* spp. and *S. nemorosa* in nectar concentration and percent monosaccharide content (Fig. 2). Pollen protein content did not differ among cultivars of any genera, but the amount of available pollen varied significantly in *Agastache* spp., *Echinacea* spp. and *Rudbeckia* spp., with no pollen produced by *R. lacianata* ‘Herbstonne’. All cultivars of *S. nemorosa* except ‘Blue Marvel’ produced no pollen, and thus analyses of pollen traits on *S. nemorosa* were excluded from subsequent analyses.

**Fig. 3.**
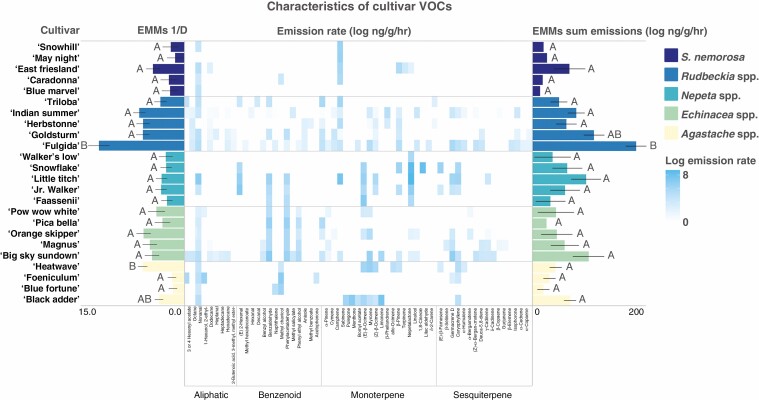
Simpson’s diversity, log emissions rate of each VOC compound, and total volatile log total emissions rate for each cultivar. Letters denote significant variation among cultivars within genera. Cultivars within some genera differ significantly in emissions rate and diversity, but most genera do not. Cultivars within many plant genera share similar VOC profiles, but some genera emit compounds unique from the rest.

Only cultivars of *Rudbeckia* spp. varied significantly from one another in the total emission rate of headspace volatiles, with ‘Fulgida’ emitting the highest amount. Cultivars of *Rudbeckia* spp. also varied significantly in the diversity of floral headspace volatiles emitted, with ‘Fulgida’ exhibiting the most complex blend and with seven unique compounds for the genus (Fig. 3). The ‘Summer Glow’ cultivar of *Agastache* spp. did not produce a detectable level of VOCs and thus was excluded from analyses.

### Floral phenotype and total visitor abundance

GLM analyses revealed a significant relationship of total pollinator abundance with corolla depth, colour, nectar composition and floral display area, although the last had the greatest positive effect (*t* = 6.09, *P* < 0.01) on total visitor abundance (see Supplementary Information for model output and analysis of residuals).

### Floral phenotype and pollinator taxonomic groups preference in the field

**Fig. 4.**
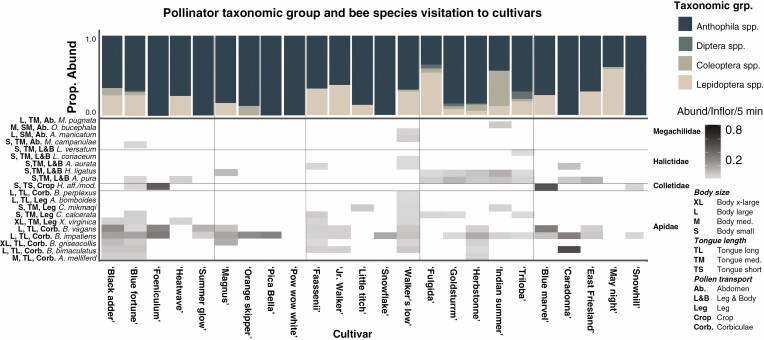
Abundance of pollinator visitors by taxonomic group and by bee species to cultivars across sites. Bee morphological traits are listed next to the species name. To avoid biasing results with ‘rare’ taxa, any taxonomic group with fewer than five total visitors to a cultivar across the whole season were removed and any bee species with fewer than four visitors to a cultivar across the whole season were removed. Bees are the primary visitors to cultivars, but cultivars vary in the bee taxa attracted and the functional traits of those species.

Across all cultivars and genera, visitors within the group Anthophila were the most abundant taxa collected at 23 out of 25 cultivars, and were the sole taxa collected on seven out of 25 cultivars. Mean abundance per inflorescence of insect taxonomic groups visiting a cultivar in a 5-min period ranged from 0.05 ± 0.01 to 0.59 ± 0.15 for Anthophila spp., 0.00 to 0.19 ± 0.11 for Coleoptera spp., 0.00 to 0.01 ± 0.01 for Diptera spp., and 0.00 to 0.46 ± 0.26 for Lepidoptera spp. (Fig. 4). Within Coleoptera, the pollen- and nectar-feeding species *Chauliognathus pensylvanicus* and *C. marginatus* were the most abundant on flowers (Riley 1892; Stugard 1931). The primary taxa within Diptera were also all flower-visiting taxa and were within the families Bombyliidae, Conopidae, Syrphidae and Tachinidae (Souza-Silva *et al.*, 2001).

### Analyses of individual floral functional traits and pollinator attraction across plant genera

#### Visual

Ordination analyses of visitor abundance/inflorescence/5 min confirmed a positive significant relationship between plant display size (PERMANOVA *R*^2^ = 0.50, *P* < 0.01, Stress = 0.022) and pollinator preference – specifically, total abundance of Anthophila spp. increased with plant height (*R*^2^ = 0.14, *P* < 0.01). Abundance of Lepidoptera spp. decreased with plant height in multidimensional space (*R*^2^ = 0.09, *P* < 0.01) but not in the direction of any cluster of plant genera, and thus this relationship is inconclusive.

Variation across genera in colour traits space, based on raw spectral data, (PERMANOVA *R*^2^ = 0.58, *P* < 0.01, Stress = 0.06) was associated with visitation rate of all pollinator taxonomic groups. Visitation by Diptera spp. and Coleoptera spp. was positively correlated in the direction of yellow flower colours (*R*^2^ = 0.36, *P* < 0.01; *R*^2^ = 0.20, *P* < 0.01) while Lepidoptera spp. and Anthophila spp. were positively correlated in the direction of purple flower colour (*R*^2^ = 0.08, *P* < 0.01; *R*^2^ = 0.14, *P* < 0.01).

**Fig. 5.**
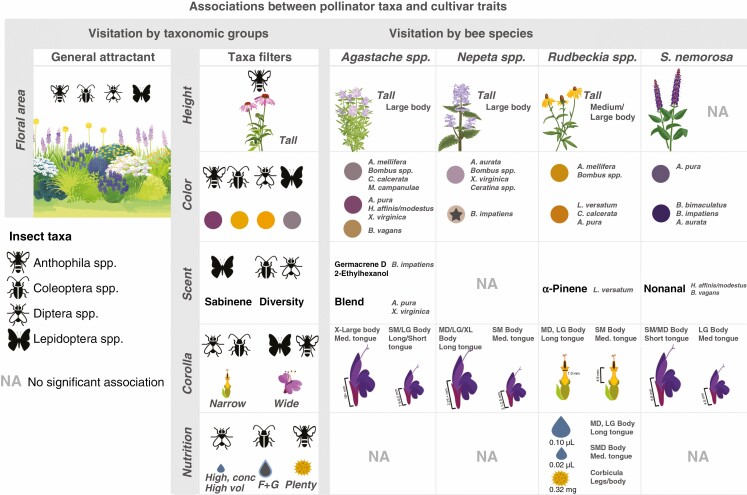
Biotic associations between pollinator taxonomic groups, bee species and floral phenotypic traits based on interpretation of linear regression and NMDS analyses. Across all plant genera, floral area acts as a general and long-distance attractant to all pollinator taxa, while traits such as height, colour, corolla morphology, nutrition and scent operate across diverse spatial scales to shape the visiting pollinator community based on insect natural history, morphology and learned associations. While these associations can be broadly used as guidelines for creating pollinator habitat in gardens, there was a high degree of generalization across pollinator taxa, particularly among bee visitors. At the level of bee species, variation in cultivar phenotype led to novel associations with some bee species and excluded others, suggesting that cultivar development can influence the attractiveness or accessibility of these varieties to a pollinator community. Furthermore, pollinator species’ response to trait variation was specific to each plant genus, supporting previous studies and demonstrating the multimodal nature of floral visual and chemical traits.

In corolla morphology space (PERMANOVA *R*^2^ = 0.71, *P* < 0.01, Stress = 0.00), visitation by Anthophila spp. and Lepidoptera spp. was positively correlated in the general direction of tubular corolla width (*R*^2^ = 0.17, *P* < 0.01; *R*^2^ = 0.01, *P* < 0.01), while visitation by Coleoptera spp. and Diptera spp. increased in the opposite direction (*R*^2^ = 0.04, *P* < 0.01; *R*^2^ = 0.28, *P* < 0.01), indicating that these taxa preferred flowers with narrower corollas (Table 1; Fig. 5).

**Table 1.** Dissimilarity of cultivars in traits space and correlation with bee visitation

	Plant Size	Colour	Corolla morphology	Nectar quality	Pollen quality	Scent emissions	Scent composition	
All genera	0.50**	0.58**	0.71**	0.35*	0.40**	0.18*	0.33**	**PERMANOVA *R*** ^**2**^
*Agastache*spp.	**0.15** ^**o**^	0.60**	0.89**	0.04	0.14	0.57^o^	0.68**	
*Echinacea*spp.	0.16	0.45**	0.04*	0.12^o^	0.07	0.06	0.13	
*Nepeta*spp.	0.52**	0.23**	0.31**	0.06	0.13	0.28	0.29	
*Rudbeckia*spp.	0.16*	0.28**	0.55**	0.33**	0.21**	0.85**	0.62**	
*S. nemorosa*	0.16	0.61**	0.87**	0.18**	*NA*	0.27	0.49**	
*Agastache*spp.	0.28**	0.42**	0.56**	0.01	0.01	−0.13	0.16^o^	**Bee species Mantel *R***
*Echinacea*spp.	0.63**	0.28**	−0.02	0.05^o^	−0.01	−0.12	−0.02	
*Nepeta*spp.	0.48**	0.15**	0.21**	0.08*	0.04	0.08	−0.02	
*Rudbeckia*spp.	0.30**	0.10**	0.41**	0.02	0.12*	0.00	0.21^o^	
*S. nemorosa*	0.13	0.37**	0.50**	0.06^o^	*NA*	−0.34	0.54^0^	
	Body size		Body size		Pollen transport			
*Agastache*spp.	0.29*		0.53**	0.00	0.03	**Fun. traits Mantel *R***		
*Echinacea*spp.	0.50*		−0.02	0.05^o^	−0.01			
*Nepeta*spp.	0.49**		0.23**	0.08*	0.04			
*Rudbeckia*spp.	0.41**		0.42**	0.05^o^	0.15**			
*S. nemorosa*	0.16^o^		0.30**	0.08*	*NA*			
			Tongue length					
		*Agastache*spp.	0.49**	0.02				
		*Echinacea*spp.	−0.02	0.05^o^				
		*Nepeta*spp.	0.22**	0.08*				
		*Rudbeckia*spp.	0.42**	0.05^o^				
		*S. nemorosa*	0.34**	0.10*				
PERMANOVA Mantel	**P **<** **0.01**	*P **≤** **0.05**	** *P* < 0.01	*P ≤ 0.05	^o^0.05 < *P* < 0.10	*P* > 0.10		
	***R*** ^**2**^ **≥** **0.15**	***R*** ^**2**^ **≥** **0.15**	*R* ^2^ ≤ 0.15	*R* ^2^ ≤ 0.15	*R* ^2^ ≥ 0.15			
	***R*** **≥** **0.15**	***R*** **≥** **0.15**	*R* ≤ 0.15	*R* ≤ 0.15	*R* ≥ 0.15			

PERMANOVA *R*^2^ values of dissimilarities of plant taxa in floral traits space and Mantel *R* values of correlations between floral traits and bee preference dissimilarity matrices. Values in bold black represent those that are statistically significant (*P* ≤ 0.05) and with a PERMANOVA *R*^2^ of at least 0.15 or a Mantel *R* of at least 0.15. Values in black, non-bold represent those that are statistically significant but that are below the determined cutoff for this study (0.15) or are close to statistically significant (0.05 < *P<* 0.10) and above the determined cutoff. Values in grey represent those that are statistically non-significant. Plant taxa vary across all plant genera in all traits except pollen nutritional qualities. Within plant genera, colour and corolla morphology are traits that are most variable across cultivars and this variation is correlated with bee species abundance and bee visitor abundance based on species’ functional traits.

#### Chemical

Dissimilarity of genera based on proportional abundance of all headspace volatile compounds (PERMANOVA *R*^2^ = 0.33, *P* = 0.01, Stress = 0.20) was relevant to abundance of Lepidoptera spp., which was positively correlated in the direction of the compounds nonanal and sabinene (*R*^2^ = 0.13, *P* = 0.01). Within phenotypic space based on floral scent emissions rate and diversity (PERMANOVA *R*^2^ = 0.18, *P* = 0.01, Stress = 0.00), visitation by Coleoptera spp. and Diptera spp. (*R*^2^ = 0.14, *P* = 0.02, R^2^ = 0.15, *P* < 0.01) was positively correlated in the direction of headspace volatiles 1/*D* and towards *Rudbeckia* spp. (Table 1; Fig. 5).

#### Nutrition

In nectar trait space across plant genera (PERMANOVA *R*^2^ = 0.35, *P* < 0.01, Stress = 0.000) (Table 1), visitation by Diptera spp. was correlated in the same direction as total nectar sugar concentration and mean nectar volume (*R*^2^ = 0.19, *P* < 0.01). Lepidoptera spp. were significantly correlated with nectar traits in traits space (*R*^2^ = 0.05, *P<* 0.01), but not in the direction of any particular traits, and thus this relationship is inconclusive in this system. Coleoptera spp. were correlated in the direction of increasing monosaccharide content (*R*^2^ = 0.09, *P* < 0.01). Abundance of Anthophila spp. was not correlated with any nectar traits in traits space.

Ordination analyses of pollen protein content and mass indicated significant dissimilarity of genera in traits space (PERMANOVA *R*^2^ = 0.40, *P* < 0.01, Stress = 0.018), largely driven by sample mass. Abundance of Anthophila spp. was correlated in the direction of pollen amount (*R*^2^ = 0.07, *P* < 0.01) while abundance of Diptera spp. and Lepidoptera spp. was correlated in the opposite direction of pollen amount (*R*^2^ = 0.05 *P* < 0.01; *R*^2^ = 0.03, *P* = 0.02) but not in the direction of any cluster of plant genera.

### Bee species visitation and cultivar-level variation in floral phenotypic traits

#### Bee species preference and bee functional traits

Following removal of ‘rare’ taxa, 20 bee species across 13 genera and four families were collected on the cultivars in this study (Fig. 4). These bee species varied from one another in the three functional traits assessed in this study: body size (ITD), tongue length and pollen transportation location. These species did not differ from one another in other functional traits that may explain variation in foraging behaviour, including phenology (Normandin *et al.*, 2017) or diet breadth, with the exception of *M. pugnata*, which is oligolectic on Asteraceae pollen (Fowler, 2016). Thus, these latter two traits were excluded from further analyses. Separate ordinations of Bray–Curtis dissimilarity matrices of the abundance of bee visitors collected at cultivars, based on taxonomic identity, revealed significant dissimilarity in bee pollinators attracted to cultivars for all genera except *Echinacea* spp. (*Agastache* spp. PERMANOVA *R*^2^ = 0.46, *P* < 0.01, Stress = 0.062; *Nepeta* spp. PERMANOVA *R*^2^ = 0.36, *P* < 0.01, Stress = 0.104; *Rudbeckia* spp. PERMANOVA *R*^2^ = 0.56, *P* < 0.01, Stress = 0.073; *S. nemorosa* PERMANOVA *R*^2^ = 0.37, *P* < 0.01, Stress = 0.070). As *Echinacea* spp. did not show any significant differences in pollinator attraction, these cultivars were excluded from subsequent analyses. Dissimilarities of cultivars based on ordinations of pollinator functional traits were overall consistent with dissimilarity based on species ordinations for all genera (Table 2).

**Table 2.** Dissimilarity of cultivars in bee preference space

	Bee species	Body size	Tongue length	Pollen transport	
*Agastache* spp.	0.46**	0.42**	0.49**	0.47**	**PERMANOVA *R*** ^**2**^
*Echinacea*spp.	0.41	0.37	0.26	0.26	
*Nepeta*spp.	0.36**	0.42**	0.54**	0.53**	
*Rudbeckia*spp.	0.56**	0.61**	0.62**	0.60**	
*S. nemorosa*	0.37**	0.35**	0.39**	0.39**	
			**P **<** **0.01** ***R*** ^**2**^ **≥** **0.15**	*P* > 0.10	

Values in bold black represent those that are statistically significant (*P* ≤ 0.05) and with and PERMANOVA *R*^2^ of at least 0.15. Values in grey represent those that are statistically non-significant. All plant genera except *Echinacea* spp. exhibited significant dissimilarity among cultivars in the bee taxa attracted, based on both species identity and functional traits.

### Correlation of bee species, bee functional traits and cultivar phenotype

Mantel tests with a Spearman correlation were performed to compare traits and pollinator preference dissimilarity matrices.

#### Visual

Dissimilarity of cultivars across all five genera in plant size traits space (Table 1; Fig. 5) was correlated with bee visitor abundance based on forager body size in *Rudbeckia* spp. (Mantel *R* = 0.41, *P* < 0.01), *Agastache* spp. (Mantel *R* = 0.29, *P* = 0.01) and *Nepeta* spp. (Mantel *R* = 0.49, *P* < 0.01), with visitation by large and medium bodied bees, particularly *Bombus* spp., increasing with plant height (Fig. 5).

Cultivars of four out of five plant genera were significantly dissimilar in floral colour. Dissimilarity of cultivars in floral colour traits space was correlated with bee species visitation to all genera (Table 1). In the three genera with purple flowers, most visitors – primarily within *Bombus* and *A. mellifera –* preferentially visited purple flowers. Notable exceptions to this were in *Agastache* spp., where visitors of *X. virginica*, *Hylaeus affinis/modestus* and *A. pura* visited the pink ‘Heatwave’ cultivar and *B. vagans* visitors increased on the light orange ‘Summer Glow’ cultivar, and in *Nepeta* spp., where *B. impatiens* increased on the white coloured ‘Snowflake’ cultivar (Fig. 5).

Dissimilarity of cultivars within all genera in corolla morphology space was correlated with bee abundance based on both species’ taxonomic identity and functional traits, but the association between corolla morphology and pollinator species’ functional traits was generally specific to each plant genus. In *Agastache* spp., small and large bodied bee species with short or long tongues preferred cultivars with moderate to long corollas (7.5–9.5 mm), while very large bee species with medium length tongues (*X. virginica*) visited cultivars with very long corollas (18+ mm). In *Nepeta* spp., corolla depth was the primary driver of bee species visitation, with long tongued and large species increasing on cultivars with deeper (<1.00 mm) corollas and all other species increasing on slightly wider corollas (1.2 mm). In *Rudbeckia* spp., long tongued bees with medium to large body size also preferred cultivars with 1.00-mm-wide corollas while small bodied species increased on cultivars with deeper corollas (4.9 mm). Finally, in cultivars of *S. nemorosa*, we observed that small and medium sized short-tongued bees preferred cultivars with longer (8.8 mm), wider (2.4 mm) corollas and larger medium-tongued bees preferred cultivars with shorter (4.5 mm) narrower corollas (1.5 mm).

#### Chemical

Three plant taxa, *Agastache* spp., *Rudbeckia* spp. and *S. nemorosa*, showed significant dissimilarities of cultivars in VOC space, based on the PERMANOVAs. Visitation of certain bee species was significantly correlated with headspace VOC emission rate and composition in traits space for these genera. However, based on Mantel tests, there was no significant association between bee preference at the community level and headspace volatile properties for these plant taxa (Fig. 5). Specifically, visitation by *Bombus impatiens* to cultivars of *Agastache* spp. was correlated in the direction of the compounds germacrene D and 2-ethylhexanol (*B. impatiens R*^2^ = 0.77, *P* < 0.01) while visitation by *X. virginica* and *A. pura* was correlated in the direction of several compounds that differentiated *A. hybrida* ‘Heatwave’ (*R*^2^ = 0.76, *P* < 0.01; R^2^ = 0.69, *P* < 0.01). For cultivars within the genus *Rudbeckia* spp., visitation by *L. versatum* was correlated in the direction of α-pinene (*R*^2^ = 0.63, *P* = 0.02). For cultivars of *S. nemorosa*, visitation by *H. affinis/modestus* and *B. vagans* was correlated in the direction of nonanal (*R*^2^ = 0.84, *P* = 0.01; R^2^ = 0.85, *P* = 0.01).

#### Nutrition

In nectar traits space, only cultivars of *Rudbeckia* spp. and *S. nemorosa* were significantly dissimilar from one another (PERMANOVA *R*^2^ = 0.33, *P* < 0.00, Stress = 0.000; PERMANOVA *R*^2^ = 0.18, *P* < 0.01, Stress = 0.000), and compared to PERMANOVA statistics of analyses on other floral traits, separation of cultivars was relatively weak (Table 1). Similar to VOC analyses, Mantel tests of the correlation of cultivars in bee preference space and nectar traits space were not significant or were below our set threshold for these genera. However, we identified significant relationships using environmental vectors. Specifically, visitation of large and medium-sized long-tongued bees increased in cultivars with higher nectar volumes in *Rudbeckia* cultivars while medium-tongued, small species preferred cultivars with lower nectar volumes. In *S. nemorosa*, small and medium sized bees increased on high-volume, low-concentration nectars but not in the direction of any cluster of cultivars and thus this relationship was inconclusive (Table 1). Only cultivars of *Rudbeckia* were significantly dissimilar in pollen traits space (PERMANOVA *R*^2^ = 0.21, *P* = 0.02, Stress = 0.000), and were significantly correlated above our Mantel test statistic threshold with pollen-carrying mechanism, with species that carry pollen either in their corbicula (*R*^2^ = 0.18, *P* < 0.01) or on their legs/body (*R*^2^ = 0.22, *P* < 0.01) associated with larger pollen amounts (Table 1).

### Validation of field data with *B. impatiens* choice assays

The probability that a single *B. impatiens* forager would select a cultivar as the first choice did not differ significantly between naive and experienced trials for all three genera tested genera except for *Agastache* spp., where experienced foragers were more likely to visit the cultivar ‘Foeniculum’ (Tukey post-hoc *P* = 0.01); however, overall preference for this cultivar was low.

**Fig. 6.**
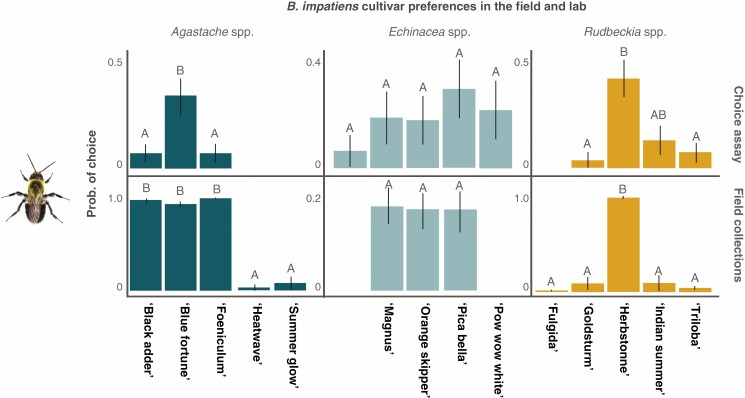
Emmeans of probability of *Bombus impatiens* visitation to cultivars in the field and in choice assays. Forager preferences in choice assays are consistent with those in the field, suggesting that variation in cultivar phenotype is relevant to species-level attraction even in the absence of community and landscape variables.

Despite potential differences in environmental conditions (ex. lighting, humidity, temperature, presence of other flowers and pollinators or lack thereof), the preferences of the bumble bee foragers were similar in both the field and laboratory assays, suggesting that the floral traits that drive these preferences are consistent, and perceived consistently, in both settings. The exception to this was in *Agastache* spp., where the cultivars ‘Black Adder’, ‘Blue Fortune’ and ‘Foeniculum’ were equally attractive in the field but ‘Blue Fortune’ was significantly more attractive than the rest in the choice assays (Fig. 6).

Comparisons of headspace volatiles 1/*D* revealed no significant differences between cut and intact flowers, but total emissions rate was significantly higher in intact vs. cut flowers. Cut and intact flowers did emit some unique compounds from one another, and a volatile (methyl jasmonate) associated with plant wounding was only found in one sample (data not shown).

## DISCUSSION

### Ornamental flowers as a tool for understanding plant–pollinator interactions

That broad patterns of visitation by pollinator taxa can be classified based on floral phenotype – or, ‘pollination syndromes’ – is a central theory in pollination ecology (Willmer, 2011). However, this hypothesis has been a topic of debate for more than a century, primarily due to the high degree of generalization in many plant–pollinator communities (Fenster *et al.*, 2004; Ollerton *et al.*, 2009). Most pollinator taxonomic groups in this study were found to be visiting plants with distinct floral phenotypes and across multiple genera, suggesting that most taxa were apparently generalized foragers (Fig. 4) and did not strictly converge on a floral trait character state (as would be expected in a more specialized relationship). Although many of the interactions between cultivars and flower visitors in this study were not apparently obligate, we still found that general patterns of visitation aligned with floral traits when considering the relative abundance of taxonomic groups on plants, probably based on pollinator physiology or natural history. For example, Coleoptera spp. lack the class of opsins that are sensitive to blue wavelengths (Sharkey *et al.*, 2017), and thus are more able to perceive white and yellow flowers. Conversely, many fly species can perceive a wide range of wavelengths and can differentiate between different coloured flowers, suggesting that their preference in this study for pale and yellow flowers may be innate (Ellis *et al.*, 2021). Thus, even in a generalized pollinator community where pollinator visitation to flowers is influenced by several biotic and abiotic factors, floral phenotype continues to be relevant in shaping the attractiveness of ornamental plants to pollinator taxonomic groups. These results demonstrate how floral phenotype and pollination syndromes may be utilized for plant selection and garden design to support a diverse pollinator community in managed greenspaces.

Our results suggest that in ornamental plants, certain floral cues, such as floral display area, may serve as general signals across taxa whereas others, such as plant height, are more taxon-specific (Fig. 5), even to the species level. For example, large-bodied bee species were positively associated with plant height in three plant genera (Fig. 5). Bee species present on these tall cultivars such as *A. mellifera* and certain *Bombus* spp. were not found to be visiting shorter varieties within these plant genera, despite being broad dietary generalists. However, tall cultivars did not preclude visitation by other bee taxa, and there were species (ex. *B. impatiens*) that were present across all or most varieties regardless of plant height (Fig. 4), suggesting that plant height may expand the visitor community to a cultivar (Thompson, 2001) (Fig. 5). Generalized opportunistic pollinator species often exhibit temporal and spatial specialization and floral constancy, particularly in communities with high plant diversity (Ebeling *et al.*, 2011). In natural systems, this hierarchical trait specialization is thought to contribute to niche partitioning at the community level (Junker *et al.*, 2013), and may be a driver of selection in generalist pollination systems. Multiple traits should be considered when developing a plant community comprising ornamental cultivars to encourage functional diversity and support temporal resource specialization across pollinator taxa.

The complex patterns of bee species visitation to cultivars illustrate that variation in floral traits at the cultivar level can influence how species interact with novel phenotypes. While in some cultivars, distinct floral traits such as plant height were related to expanding the visiting pollinator community (see above), in others, trait variation led to the exclusion of potential visitors. We observed that purple-coloured cultivars within genera were widely visited by many bee taxa, while non-purple cultivars supported only a limited subset of the bee pollinator community. Specifically, certain *Bombus* species were present on all or most cultivars within *Nepeta* spp. and *Agastache* spp. but were the sole visitors to *N. racemosa* ‘Snowflake’ (*B. impatiens*) and *A. hybrida* ‘Summer Glow’ (*B. vagans*, *B. impatiens*) (Fig. 4). Indeed, *Bombus* spp. are highly generalized and are quite adept at learning novel flower signals to exploit rewards (Gumbert, 2000), and therefore may be particularly flexible in overcoming the limitations of variation in cultivar colour. Additionally, we found that variation in corolla depth and width directly affected visitation of bee species to cultivars, indicating mechanical exclusion based on bee functional morphological traits (Wester *et al.*, 2020) (Fig. 5). Namely, pollinator visitation to *Agastache* cultivars with very long corollas (‘Summer Glow’ and ‘Heatwave’) was correlated with bee tongue length. Within *Agastache* spp., the ‘Summer Glow’ corolla was on average 3 mm shorter (~18 mm) than that of ‘Heatwave’ (Fig. 2), and thus nectar rewards were probably accessible to long-tongued *Bombus* spp. Conversely, the sole visitors to ‘Heatwave’ (21-mm corolla) were large-bodied *X. virginica* spp., which were often observed to be nectar robbing, and small-bodied *A. pura*, which were observed to be accessing nectar rewards by travelling inside the corolla. Our results have implications for understanding both how cultivar breeding may influence plant utility to pollinators and how trait variation in wild plant populations can influence pollinator visitation (ex. Bradshaw and Schemske, 2003) and drive adaptive evolution in a generalized community.

The lack of an association between bee species attraction and plant nutritional traits to most taxa in this study was somewhat surprising, both because nectar properties were significant drivers of visitation rates across the broader taxonomic groups and pollen nutritional composition often promotes specialized foraging behaviour, even in generalist bee taxa (Roulston and Cane, 2000; Vaudo *et al.*, 2016). These results suggest that the differences in nectar and pollen quality and quantities in the cultivars in our studies were not sufficiently large enough to drive differences in visitation patterns in the field (Heinrich, 1979; Keasar *et al.*, 2008; Lemaitre *et al.*, 2014; Nepi *et al.*, 2018), though clearly breeding can reduce nutritional resource availability in many cultivars (Comba *et al.*, 1999). Alternatively, other factors that were not evaluated in our analysis, such as floral microbial communities (Russell and McFrederick, 2021) or varying rates of evaporation due to daily variation in environmental conditions in the field, could have influenced nutritional quality and quantity (Pleasants, 1983).

### Practical applications to plant breeding

The results of this study can assist ornamental flower breeders in identifying key heritable traits that affect pollinator attraction as well as those traits that are less significant. For example, flower colour, morphology and floral display area are all traits that respond readily to artificial selection and are commonly selected for in cultivar development (Mol *et al.*, 1995; Comba *et al.*, 1999; Yang *et al.*, 2019), and were the traits that consistently were related to pollinator visitation in this study. Our results indicate that plant breeding and artificial selection can have direct effects on the attractiveness or accessibility of cultivars to pollinators, although further studies are needed to explore this hypothesis.

Floral traits, such as headspace volatiles, can evolve rapidly under selection (Zu *et al.*, 2016), and the biosynthetic pathways that build these compounds are often located on the same chromosomes as those that regulate floral pigment deposition (Raguso *et al.*, 2015) or flower structural characteristics such as corolla length (Smith, 2016). Thus, cultivar development and plant breeding may also influence plant attractiveness to pollinators through indirect selection on phenotypic traits. In this study, there was variation at the cultivar level in floral scent traits for some genera studied, in particular in the individual compounds produced (Fig. 2), which was probably not a product of direct artificial selection and breeding. Thus, this intercultivar variation in traits such as floral headspace volatiles may indicate pleiotropic consequences of direct selection on traits such as floral colour (Sobel and Streisfeld, 2013), morphology or plant stature (Zu and Schiestl, 2017). However, the genera with the greatest variation in floral VOCs were also those that comprised cultivars from distinct parent species, which may indicate that in this system, dissimilarity of cultivar VOC emissions and composition may largely reflect the parental genotypes of hybrid varieties (Stearn, 1950).

While it is assumed that cultivar development in ornamental flowers would uncouple floral traits from nutritional quality, these results suggested that the nutritional content and quality of the flowers in this study are apparently intact (Wright and Schiestl, 2009). In choice assays, naive and experienced *B. impatiens* foragers showed largely the same preferences among cultivars as we recorded in field studies. This suggests that for these ornamental genera, floral visual and chemical advertisements remain sufficiently honest despite variability in character state (Knauer and Schiestl, 2015), or that the nutritional rewards provided by the different cultivars were sufficiently equivalent and ‘average’ to support this generalist pollinator species (Fig. 4).

## CONCLUSION

This study illustrates the value of ornamental cultivars as a model to explore the mechanisms of plant–pollinator interactions across multiple scales. Moreover, understanding how variability in cultivar phenotypic traits influences plant attractiveness, accessibility and nutritional quality to different pollinator taxa has valuable applications for stakeholders.

Identification of single traits or suites of floral traits that either exclude or encourage pollinator taxonomic groups can help inform plant selection in domestic pollinator habitats, from home gardens to urban greenspaces. We demonstrate that at the level of the total ecological community, increasing total flowering plant density is one of the most reliable methods for bringing diverse pollinator taxa, particularly generalist species, into a habitat (Laha *et al.*, 2020; Staab *et al.*, 2020). Furthermore, at the level of pollinator taxonomic group, associations between floral traits and pollinator preference were generally consistent with previously published studies of plant–pollinator interactions. Thus, at a broad scale, patterns of preference in generalist pollinators are largely conserved and floral phenotype may be used as a general guideline for informing cultivar selection for managed pollinator habitats. As pollinator species and taxonomic groups exhibited distinct patterns of attraction to floral visual, chemical and nutritional traits, managing a habitat with a broad selection of plants and phenotypes will be necessary for supporting a species-rich and functionally diverse pollinator community (Normandin *et al.*, 2017; Kremen *et al.*, 2018). Furthermore, while bee species are broadly attracted to these cultivars, there are clear species-level differences in their preferences to different floral traits. Thus, phenotypic variation among cultivars can predict differential attraction of bee taxa based on species identity and functional traits and lend insight into the implications of artificial selection and plant breeding.

Our results demonstrate that there is substantial flexibility in the role of traits for shaping pollinator attraction to ornamental plant cultivars. In some cases, cultivar development leads to novel interactions with pollinator taxa, meaning that cultivars may support niche differentiation in modified landscapes and increase community functional diversity. In other instances, cultivar development may exclude taxonomic groups, resulting in overall low utility of that variety to a community. Furthermore, direct artificial selection on floral phenotype may have pleiotropic effects on expression of other behaviourally significant traits, such as emissions of floral headspace volatiles, which should be considered when developing new cultivars to support pollinators. Therefore, the ornamental plant breeding industry has tremendous potential and power to develop cultivars that can support either specific groups of pollinators or a diversity of taxa.

## SUPPLEMENTARY DATA

Supplementary information are available online at https://academic.oup.com/aob and consist of the following. Details on any relevant chemical treatments of study plants obtained from the nursery and information on vernalization of study plants. We outline further methods on VOC collection and identification, nectar and pollen analysis, and the *Bombus* foraging arena specifications for choice assays. We also include a detailed description of the model parameters used to analyse floral traits data. Figure S1 is an example of our foraging arenas and Table S1 has information on colony-level replication for choice assays. Table S2 contains information on replication and collection methods of floral traits. Table S3 contains information on bee functional traits and the categorical traits used in these analyses. We also include a section on statistical results, with data from PERMANOVA tests of repeated measures on biological replicates, model output from the GLM using all floral traits as predictors and total visitor abundance as the response variable, and DHARMa analysis of residuals (Fig. S2). Finally, we report which cultivars exhibited variation in nutritional traits across years and sites based on LMMs.

mcac082_suppl_Supplementary_Material
