# Supplementary material for: Complex floral traits shape pollinator attraction to ornamental plants
Source: Ann Bot. 2022 Jun 22;130(4):561–77. doi: 10.1093/aob/mcac082 (PMC9510942; doi:10.1093/aob/mcac082)
Supplement: mcac082_suppl_Supplementary_Material [file mcac082_suppl_supplementary_material.docx]

**Complex floral traits shape pollinator attraction to ornamental plants**

Erickson, E.*^1^, Junker, R. R.^2,3^, Ali, J.G^1^, McCartney, N.^1^, Patch, H.M.^1^, Grozinger C.M.^1^

**SUPPLEMENTARY MATERIAL**

**Methods**

***Treatment of plant plugs prior to study***

We received the following information on which chemicals plugs were treated with at North Creek Nurseries, where the vast majority of our plant material was sourced: Hydrogen peroxide, peroxyacetic acid, octanioic acid, Cease/Milstop : Bacillus subtillus strain QST 714, Potassium bicarbonate, Medallion : Fludioxinil, Strobe : Azoxystrobin, Pageant/X3 : Pyraclostrobin/boscalids and Hydrogen peroxide, peroxyacetic acid, octanioic acid

***Vernalization of greenhouse plants***

To ensure proper plant growth, all potted plants were vernalized outdoors from 11/25/2019 until 1/20/20 where the average daily temperature was between 10.5 °C and -2.75 °C (data from climate.psu.edu). Less cold-hardy cultivars were covered with a frost protection blanket (Planket, Dallas TX) to increase overwinter survival.

***VOCs measurements in cut flowers***

Push-pull collections were performed on 3 replicates/cultivar of cut flowers of *Rudbeckia* spp., *Agastache* spp., and *Echinacea* spp. in a climate-controlled growth chamber to assess whether cut flowers, which were used for *B. impatiens* choice assays, had unique floral scent bouquets. Flowers were cut immediately preceding VOC collection and were stored in pics with fresh water throughout collection. Each flower was placed within a glass chamber (20 x 30 cm) on teflon bases. Air was pushed through an activated charcoal filter at a flow rate of 250ml/min and VOCs were collected over HayeSepQ filter traps (Sigma Aldrich, USA). Collections ran for 3 hours, consistent with the maximum time after removal from the whole plant that a cut flower replicate was used in *Bombus* behavioral assays.

***VOC identification***

Compounds were first in ChemStation (Agilent, USA) with >90% confidence using the Adams mass spectral library. Library identifications below an 80% quality match and that were greater than 5% difference from the highest quality match for each peak were removed. Identification of VOCs for each sample was confirmed using a combination of manual spectral matching and comparison of spectral-retention indices to alkane standards using the Adams mass spectral-retention index library. Characteristics of compounds were classified based on Knudsen *et al.* 2006. All compounds that were only observed on one of the three replicates were omitted from statistical analysis.

***Nectar analysis***

Serial diluted standards of known concentrations were run alongside samples for each analysis event and results were only used if the R value for the standard curve was at least 0.90. For analysis, I calculated the mean volume per floret, the total concentration (%w/w) and the percent monosaccharide (Fructose + glucose / total concentration *100) content for each replicate.

***UHPLC methods for nectar analysis***

Nectar samples were analyzed using the following four parameters in UHPLC:

A: 0.5ml/min 80%ACN, column compartment at 35°C RI at 35°C, 2.5ul injection volume

B: 1.0ml/min 85%ACN, column compartment at 40°C RI at 35°C, 3ul injection volume

C: 0.5ml/min 85%ACN, column compartment at 35°C RI at 35°C, 3ul injection volume

***Pollen analysis***

To remove pollen from anthers, entire samples were transferred to modified spin filter tubes fitted with 100-micron stainless steel mesh. Samples were lyophilized (Labconco FreeZone 1177030, Kansas City MO) for three hours at -80°C to remove all moisture. Samples were then agitated using a bead mill homogenizer (OMNI International Lenesaw, GA) at 4.0 m/s in four 30 second increments to extract pollen grains from the entire samples. Samples were centrifuged for five minutes at 13000 RPM to separate pollen from non-pollen plant tissue, which was subsequently discarded. Pollen samples were then weighed into two separate vials (0.005 mg – 1.2 mg), one vial for use in this protein analysis and one for a lipid analysis that was ultimately not included in this study. Samples were measured using a microbalance (Mettler Toledo XPE26) and the weight of each sample was recorded. For samples that exceeded 2.4 mg (the maximum sample weight for pollen nutritional analyses), the remaining sample was weighed, and this mass was included in the estimate of total pollen availability.

***Foraging arena specifications***

Foraging arenas (90x54x46 cm) painted a neutral matte grey on the bottom and three sides (Rustoleum #285143). One side of each arena was made with plexiglass to allow for observation. The plexiglass side also had an inlet hole to allow for attachment of the colony. The top of each arena was made of a light diffusing panel and an LED light strip was suspended at 0.5 meter above the arenas.

***Statistical analysis - linear models and linear mixed effects models of all floral traits***

For all measured floral traits, the trait measurement was set as the response variable and cultivar (subset by plant genus) was set as the fixed effect. For traits with repeated measures (petal color, nectar properties, pollen properties, and corolla morphology), plant replicate was set as the random effect. For floral display area, both plant replicate and site were set as the random effects and for plant height, site was set as the random effect.

**Lab choice-assay foraging arena**

**
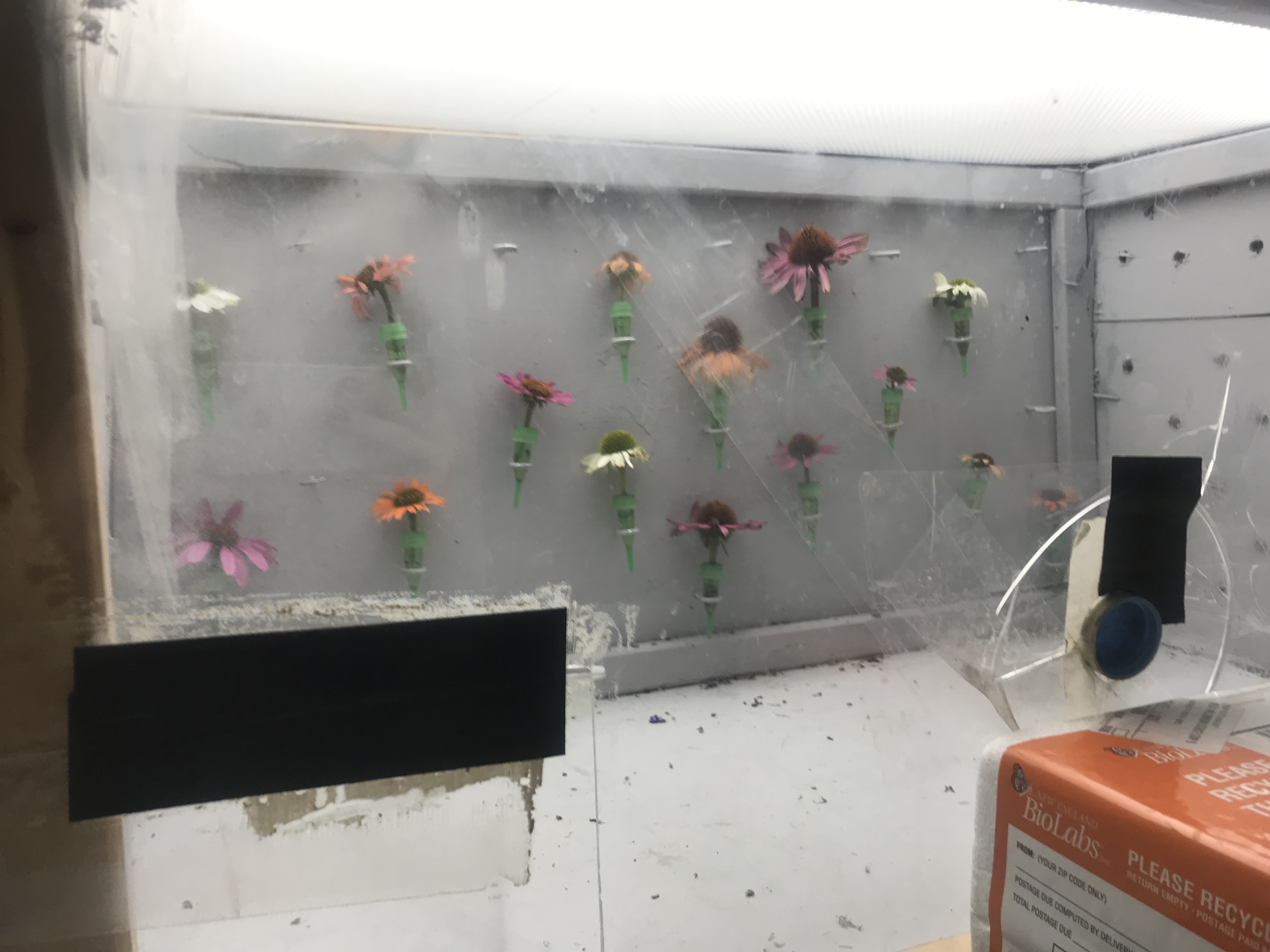
**

**Figure S1.** An example of the testing arenas used in *B. impatiens* foraging assays. For these assays, 3 replicates of each cultivar within plant genera were mounted on the back wall of a custom built arena. Foragers were allowed to fly freely and foraging behavior of flower-naive and experienced foragers was recorded. A separate arena (not shown) was used to train foragers to fly and gather resources in the test arena.

|  | **Colony A** | **Colony B** | **Colony C** | **Colony D** | **Colony E** | **Colony H** |
| --- | --- | --- | --- | --- | --- | --- |
| **Agastache** | 7 | 3 | 6 | 6 | 3 | *NA* |
| **Echinacea** | 6 | 6 | 6 | 6 | *NA* | *NA* |
| **Rudbeckia** | 6 | 1 | 3 | 6 | *NA* | 6 |

**Table S1.** **Number of *B. impatiens* choice trials per colony and plant genus**

**Table S2. Methods and replication of floral traits data collections**


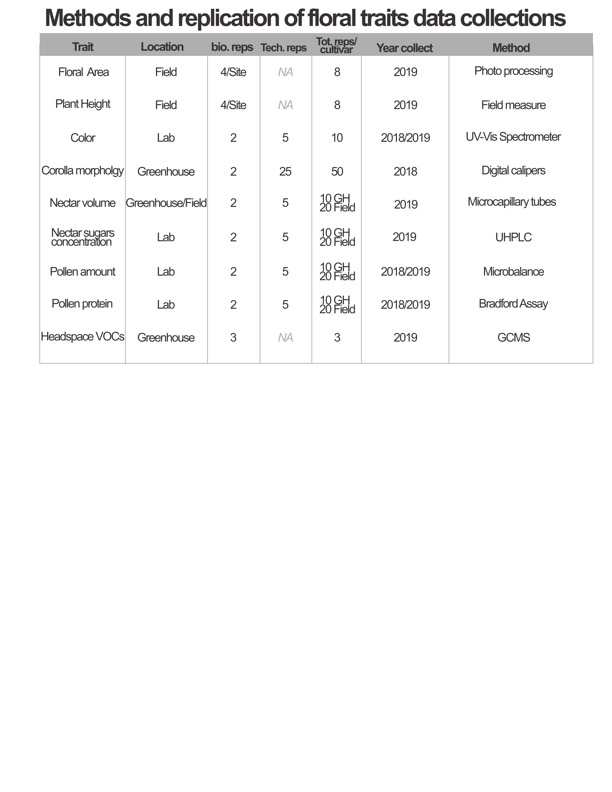


**Table S3. The relevant functional traits of the dominant bee species collected in this study**

| Family | Genus | Species | ITDMM | O_P | Pollen_transport | Body Size | Tongue_length | Source |
| --- | --- | --- | --- | --- | --- | --- | --- | --- |
| Colletidae | Hylaeus | affinis/modestus | 1.244 | Polylectic | Crop | Small | Short | Bartomeus 2013; Normandin 2017 |
| Apidae | Ceratina | calcerata | 1.38 | Polylectic | Legs | Small | Medium | Bartomeus 2013; Normandin et al. 2017 |
| Halictidae | Lasioglossum | versatum | 1.43 | Polylectic | Leg&Body | Small | Medium | Bartomeus et al. 2017.; Normandin 2017 |
| Apidae | Ceratina | mikmaqi | 1.534 | Polylectic | Legs | Small | Medium | Normandin et al. 2017 |
| Halictidae | Augochlorella | aurata | 1.654 | Polylectic | Leg&Body | Small | Medium | Bartomeus 2013; Normandin et al. 2017 |
| Halictidae | Halictus | ligatus | 1.982 | Polylectic | Leg&Body | Small | Medium | Bartomeus 2013, Normandin et al. 2017 |
| Halictidae | Augochlora | pura | 2.198 | Polylectic | Leg&Body | Small | Medium | Bartomeus 2013; Normandin et al. 2017 |
| Megachilidae | Megachile | campanulae | 2.26 | Polylectic | Abdomen | Medium | Medium | Bartomeus et al. 2017.; Normandin 2017 |
| Halictidae | Lasioglossum | coriaceum | 2.59 | Polylectic | Leg&Body | Medium | Medium | Bartomeus 2013; Normandin 2017 |
| Megachilidae | Anthidium | manicatum | 3.322 | Polylectic | Abdomen | Medium | Medium | Normandin et al. 2017 |
| Apidae | Anthophora | bomboides | 3.518 | Polylectic | Legs | Medium | Long | Rykken 2017 |
| Megachilidae | Osmia | buscephala | 3.7 | Polylectic | Abdomen | Medium | Medium | Bartomeus 2013; Normandin et al. 2017 |
| Apidae | Bombus | perplexus | 3.79 | Polylectic | Corbiculae | Medium | Long | Bartomeus 2013; Normandin et al. 2017 |
| Apidae | Bombus | vagans | 3.804 | Polylectic | Corbiculae | Medium | Long | Bartomeus 2013; Normandin et al. 2017 |
| Megachilidae | Megachile | pugnata | 4.3 | Oligolectic | Abdomen | Medium | Medium | Bartomeus et al. 2017.; Normandin 2017 |
| Apidae | Bombus | bimaculatus | 4.398 | Polylectic | Corbiculae | Large | Long | Bartomeus 2013; Normandin et al. 2017 |
| Apidae | Bombus | impatiens | 4.852 | Polylectic | Corbiculae | Large | Long | Bartomeus 2013; Normandin et al. 2017 |
| Apidae | Xylocopa | virginica | 6.848 | Polylectic | Legs | X_Large | Medium | Bartomeus 2013; Normandin et al. 2017 |
| Apidae | Bombus | griseocollis | 7.4 | Polylectic | Corbiculae | X_Large | Long | Bartomeus 2013; Normandin et al. 2017 |

**Statistical results**

***PERMANOVA results for repeated measures - traits collections***

**All genera**

adonis(formula = distance ~ Replicate, data = data, method = "euclidean" strata = data$plantID)

**Color**

Replicate: R^2^ = 0.00, *P* = 0.1

**Corolla Morphology**

Replicate: R^2^ = 0.00, *P* = 0.62

**Nectar**

Replicate: R^2^ = 0.01, *P* = 0.17

**Pollen**

Replicate: R^2^ = 0.02, *P* = 0.66

***Results of GLM for all floral traits and total visitor abundance***

*Equation*

glm(formula = total_viz ~ log(Height_cm) + log(Area_cm2) + Site + Hue_angle +

mouth + depth + volume + conc + per_mono + log(voc_emission) +

voc_diversity + pollen_prot + poll_mass + PresenceUV, family = Gamma(link = "log"),

data = m4_fin3)

Deviance Residuals:

Min 1Q Median 3Q Max

-1.2228 -0.4655 -0.1343 0.2770 1.0783

| Coefficients: | Estimate | Std. Error | t value | Pr(>\|t\|) |
| --- | --- | --- | --- | --- |
| (Intercept) | 7.41590 | 3.24602 | 2.28461 | 0.02551 |
| log(Height_cm) | -0.59777 | 0.73602 | -0.81217 | 0.41957 |
| log(Area_cm2) | 1.89944 | 0.31184 | 6.09112 | 0.00000 |
| SiteSwitch | 0.15902 | 0.14884 | 1.06836 | 0.28919 |
| Hue_angle | -0.00418 | 0.00192 | -2.17753 | 0.03297 |
| mouth | 0.62193 | 0.37558 | 1.65595 | 0.10241 |
| depth | -0.25898 | 0.09190 | -2.81820 | 0.00634 |
| volume | -0.80812 | 0.79662 | -1.01443 | 0.31403 |
| conc | -0.07669 | 0.04652 | -1.64861 | 0.10391 |
| per_mono | -0.07290 | 0.02521 | -2.89159 | 0.00516 |
| log(voc_emission) | 0.23233 | 0.24281 | 0.95681 | 0.34210 |
| voc_diversity | 0.14508 | 0.12641 | 1.14764 | 0.25520 |
| pollen_prot | -0.00462 | 0.00465 | -0.99450 | 0.32356 |
| poll_mass | -0.43786 | 0.33380 | -1.31174 | 0.19409 |
| PresenceUV | 0.10275 | 0.29391 | 0.34960 | 0.72774 |

***DHARMa output***


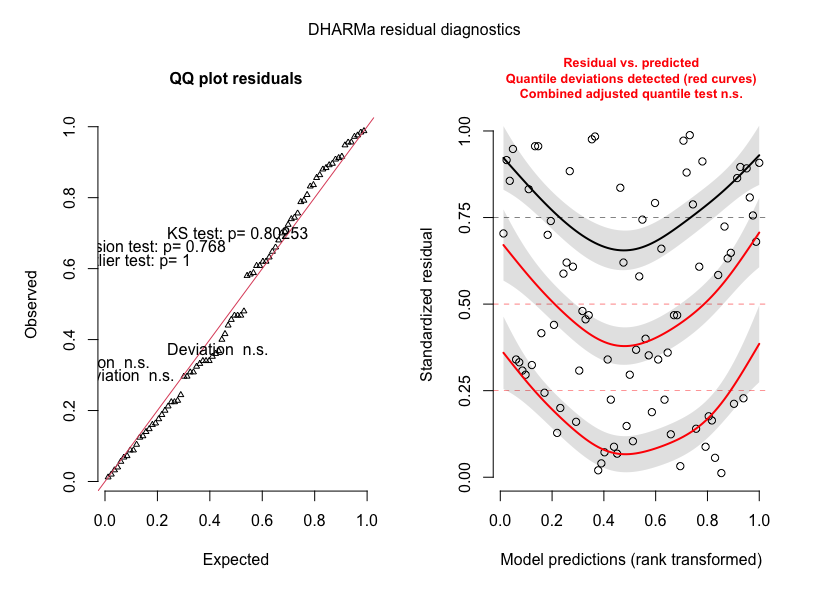


**Figure S2.** Residuals analysis in DHARMa revealed some deviation of the quantiles (*P* = 0.06) but no significant deviation in QQplots. Given then QQ plot and the consistency of the quantile patterns, we conclude that our model has suitable fit.

**Cultivars that differ in pollen and nectar properties between sites from Linear Mixed Effects Models**

***SITE***

***Nectar volume***

*Salvia nemorosa* 'Blue Marvel' *P* = 0.04

*Salvia nemorosa* 'East Friesland' *P* = 0.05

***Nectar concentration***

*Salvia nemorosa* 'Blue Marvel' *P* = 0.03

*Salvia nemorosa* 'East Friesland' *P* = 0.02

*Salvia nemorosa* 'May Night' *P* = 0.03

***Nectar monosaccharide percentage***

*Agastache foeniculum* 'Foeniculum' *P* < 0.01

*Agastache hybrida* 'Summer Glow' *P* = 0.03

*Echinacea purpurea* 'Magnus' *P* = 0.03

*Rudbeckia hirta* 'Indian Summer' *P* = 0.01

***Pollen protein***

*None*

***Pollen mass***

*Echinacea purpurea* 'Pica Bella' *P <* 0.01

***YEAR***

***Nectar volume (Site 2 only)***

*No significant difference*

***Nectar concentration (Site 2 only)***

*Echinacea hybrida* 'Big Sky Sundown' *P* < 0.001

*Rudbeckia hirta* 'Indian Summer' *P* < 0.01

*Rudbeckia triloba* 'Triloba' *P* < 0.01

***Nectar monosaccharide percentage (Site 2 only)***

*Agastache foeniculum* 'Foeniculum' *P* = 0.01

*Salvia nemorosa* 'Caradonna' *P* < 0.01

***Pollen protein***

*Rudbeckia hirta* 'Indian Summer' *P* = 0.02

*Nepeta racemosa* 'Little Titch' *P* = 0.03

***Pollen mass***

*Echinacea purpurea* 'Magnus' *P* < 0.01

*Echinacea purpurea* 'Pica Bella' *P* = 0.03
